# Combined treatment with intranasal esketamine and electroconvulsive therapy in resistant depression. Two cases report

**DOI:** 10.1192/j.eurpsy.2025.1315

**Published:** 2025-08-26

**Authors:** P. Del Sol, M. García Moreno, L. Gayubo Moreo, A. Izquierdo de la Puente

**Affiliations:** 1 Psychiatry, Hospital Puerta de Hierro, Madrid, Spain

## Abstract

**Introduction:**

Depressive symptoms that do not respond to 2 lines of antidepressant treatment in adequate doses for 6-8 weeks are known as resistant depression. As therapeutic alternatives we currently have, among other options, intranasal esketamine and electroconvulsive therapy (ECT).

**Objectives:**

To present two cases of resistant depression in combined treatment with esketamine and ECT as an effective tratment

**Methods:**

2 cases report

**Results:**

The first one is a 60-year-old male with a diagnosis of recurrent depression who was admitted after an autolytic attempt by drug overdose. Our second patient is a 59-year-old male with a diagnosis of bipolar disorder, current major depressive episode with psychotic symptoms. He had a history of previous depressive episodes, requiring treatment with ECT on 2 occasions due to resistance to psychopharmacological treatment.

Both patients had major depressive symptoms resistant to conventional pharmacological treatment, with a predominance of sadness, apathy, anhedonia, hopelessness, psychomotor inhibition and self-induced suicidal ideation. One of them also presented psychotic symptoms congruent with mood.

The first patient received treatment with intranasal esketamine with partial response, so a combination with ECT was started once the 8 biweekly sessions of the induction phase were completed. The second patient was initially ambivalent to a new cycle of ECT. For this reason, treatment with esketamine was proposed and after 6 biweekly sessions he agreed to overlap treatment with ECT. In both patients there was a clear improvement in clinical symptoms and adequate tolerability, allowing discharge home.

**Conclusions:**

There are few data in the literature on combined treatment with intranasal esketamine and ECT. Our experience in the 2 cases described points to an adequate response and tolerability. Specific studies would be necessary in this regard.

**Disclosure of Interest:**

None Declared

